# The King and the Chichester Infirmary

**Published:** 1913-03-08

**Authors:** 


*To the Editor of* The Hospital.

### The King and the Chichester Infirmary.

*To the Editor of* The Hospital.

Sir,—I have read a comment in your last issue *re* the Chichester Infirmary, and the fact that His Majesty the King has graciously consented to open the reconstructed building on August 2; and you also comment on the plan which has been adopted for paying off the debt, and hope "that the small remaining sum will be raised without the necessity of realising investments." I would like to explain that it will be quite unnecessary to realise any investments to pay off the debt.

The trustees have in hand a sum, which has been received from legacies, nearly sufficient to pay off the debt, and which is not invested, but is lying on deposit at the bank. The board have been informed that a further legacy will be paid in very shortly.

This legacy far exceeds the amount of the debt.

It was necessary to get permission from the governors at the annual general meeting to use this money, as a rule has been adopted in this institution that all legacies go automatically to the trustees' account, and can only be transferred to the general account if sanctioned by the governors at a general meeting.

The board are making every effort to place the funds on a sound footing by raising sufficient income each year to meet the expenditure and investing all legacies. In 1911 success crowned their efforts, the income exceeding the expenditure by about £70, without touching the legacies. This, I think you will agree with me, is sound finance.

Last year (1912), owing to a large falling off in donations from money-boxes, penny-a-month fund, Church offertories, etc., and a slight falling off in annual subscriptions, which were caused, to a very large extent, by the Insurance Act, there was a deficit on the year's work of £391; but we have every hope that in 1913 the income and expenditure will again be made to meet.

The debt which it is proposed to pay off was caused by (1) the loss in 1912, and (2) by a change in the system of accounts which was carried into effect about three years ago.

Bills which were paid quarterly it was decided to pay monthly, and the date for receiving annual subscriptions was altered from September 1 to January 1. This, of course, caused a heavy loss at the time, but it will not occur again. About 75 per cent. of the debt is really due to reorganisation, and has nothing to do with the ordinary income and expenditure, and is, I think, a fair charge against the trustees' account, while the other 25 per cent. would probably not have occurred if it had not been for the Insurance Act.

I hope this explanation will make the matter quite clear.—Yours faithfully,

A Member of the Board.

Chichester, February 23, 1913.

